# Curators of the Medical Department of the University of Buffalo

**Published:** 1851-07

**Authors:** 


*Curators of the Medical Department of the University of Buffalo.*—The following gentlemen have been recently elected by the Council of the University of Buffalo, Curators of the Medical Department:

| | |
|---|---|
| Dr. E. Wallis, of Buffalo. | Dr. Samuel Carey of Buffalo. |
| " S. F. Mixer, " | " George P. Eddy, Lewiston, |
| " J. B. Samo, " | " Orlando Wakely, Clarence. |

Dr. M. G. Lewis, Black Rock.

